# Machine learning-based prediction of cross-immunity

**DOI:** 10.1093/bib/bbag484

**Published:** 2026-09-10

**Authors:** Vivien Erzsébet Resch, László Tóth, Anita Rácz, Dezső Virok, Gábor Paragi

**Affiliations:** Department of Medicinal Chemistry, University of Szeged, Dóm tér 8, H-6720 Szeged, Hungary; Institute of Physics, University of Pécs, Ifjúság útja 6, H-7625 Pécs, Hungary; Institute of Informatics, University of Szeged, Árpád tér 2, 6720 Szeged, Hungary; Plasma Chemistry Research Group, HUN-REN Research Centre for Natural Sciences, Magyar Tudósok Körútja 2, H-1117 Budapest, Hungary; Department of Medical Microbiology, Albert Szent-Györgyi Health Center and Albert Szent-Györgyi Medical School, University of Szeged, Semmelweis Str. 6, H-6725 Szeged, Hungary; Department of Medicinal Chemistry, University of Szeged, Dóm tér 8, H-6720 Szeged, Hungary; Institute of Physics, University of Pécs, Ifjúság útja 6, H-7625 Pécs, Hungary; Department of Theoretical Physics, University of Szeged, Tisza L. krt. 84-86, H-7625 Pécs, Hungary

**Keywords:** TCR–pMHC activation, machine learning, peptide representations, consensus models of representations, cross immunity

## Abstract

Cross-immunity, defined as the ability of T-cells to recognize multiple antigen peptide-major histocompatibility complexes, is a fundamental feature of adaptive immunity. However, the prediction of different peptide epitopes that can be recognized by the same T-cell receptor remains challenging. Currently, artificial intelligent (AI)-based machine learning (ML) methods can be successfully used for pattern recognition in epitope molecular space by detecting the functional similarity between peptide sequences. In this study, using literature-based experimental data, we examined ML-based binary classification models trained on small datasets to predict the activity of nine-amino-acid-long peptides. Our results suggest that the consensus function of well-established similarity matrix-based representations and structural-based descriptors of epitopes yields better performance because representation-specific noises are reduced and individual model weaknesses are partially compensated. We also sought to determine the extent to which the predictive power of the applied AIs procedure depended on the physicochemical content of the descriptor set during the training process. In addition, challenging the models, we applied them to an independent experimental dataset to examine the effects of diverse laboratory conditions on a regulated biological measurement. In summary, applying a consensus function can capture the biological complexity of cross-reactivity at the binary classification level, even when applied to relatively small datasets.

## Introduction

The immune system comprises of a complex network of cells and molecules. Among the key players, T-cells play a crucial role in adaptive immunity, providing specific and long-lasting defense against intracellular pathogens and cancer cells. A fundamental step in T-cell activation is the identification of the peptide-major histocompatibility complex (pMHC) on target cells or professional antigen-presenting cells by the T-cell receptor (TCR). A subset of T-cells, CD8+ cytotoxic T-cells, recognize antigens presented on MHC Class I molecules (MHC-I), and upon activation, directly kill infected or cancer cells. It is well known that antigen epitopes complexed with MHC-I are predominantly 9-mers [1]. An interesting discrepancy is that the estimated functional TCR repertoire set (~10^7^–10^8^) is significantly smaller than the potential number of possible 9-mer epitopes (on the order of 10^11^) [2]. Therefore, cross-reactivity, i.e. the ability of a single TCR to recognize multiple pMHC complexes, can be a frequent event [3]. T-cell polyspecificity or T-cell cross-immunity plays a significant role in various scientific and medical applications, such as vaccine development and cancer immunotherapy, especially off-target effects of targeted T-cell therapy and understanding of autoimmunity [4]. The TCR antigen-binding site, formed by the variable regions of the alpha (α) and beta (β) chains [or the gamma (γ) and delta (δ) chains in γδ T-cells], interacts with the common molecular surface formed by peptide and MHC proteins. This interaction involves multiple contact points, and variations in the peptide sequence can still allow sufficient binding affinity to trigger T-cell activation [5]. Because of the recognition mechanism, highly similar epitope sequences differing only by a single amino acid may not be recognized by the same TCR. In contrast, epitopes with multiple differences in sequence but similar physiochemical characteristics can elicit similar immune responses. Prediction of peptide epitope binding to a particular MHC is well described, and various software tools are available for this task such as NetMHCpan [6], MHCSeqNet2 [6], and PickPocket [7]; however, the prediction of cross-immunity is less characterized. CrossDome [8] calculated the sequence similarity between known epitopes and potential cross-reactive peptides and ranked potential cross-reactive epitopes, without any physicochemical characterization or geometrical information. PepSim [9] used the structural information of TCR–pMHC complexes to model potential interactions with novel peptide sequences.

Machine learning (ML) methods enable the discovery of complex patterns and relationships within vast biological datasets, leading to new predictive capabilities that traditional analytical methods cannot achieve. Although deep learning techniques such as large neural network models typically require extensive datasets for effective training and prediction, some methods can be successfully applied to smaller datasets comprising only a few hundred instances [10]. Recently, Human Leukocyte Antigen (HLA)-A*02–peptide TCR crystal structures were examined using a multimodal geometric deep-learning framework incorporating an immunological-fingerprint strategy [11]. This approach enables systematic decoding of structural cues that underlie TCR recognition, including critical spatial features governing antigen engagement.

In this work, we adopted machine-learning techniques on a literature-derived dataset of T-cell activation. Our goal was to develop a computational procedure capable of predicting the activity of MHC-I epitopes [12]. The proposed approach is expected to effectively distinguish between active and inactive 9-mer peptides, and its predictive performance has been validated using an external dataset [13]. Furthermore, we compared the performance of a small-scale feedforward neural network (FFNN) [14] with three widely used conventional ML classifiers: random forest (RF) [15], logistic regression (LR) [16], and support vector machine (SVM) [17] methods. These models were selected because they represent distinct modeling strategies and therefore provide a broader assessment of classifier performance. FFNN is a neural-network-based model characterized by unidirectional information flow through successive layers without feedback connections. This architecture allows nonlinear relationships between the input features and output classes to be learned and is widely used in classification and pattern-recognition tasks [18]. In contrast, RF is a non-parametric ensemble method that combines multiple decision trees to improve predictive stability and reduce variance. It can also capture nonlinear feature interactions, and is commonly applied to small and medium-sized biological datasets. The LR was included as an interpretable probabilistic baseline model. It estimates the probability of class membership using a linear decision function, and serves as a useful reference for assessing whether more complex nonlinear models offer additional predictive benefits. The SVM was included as a margin-based classifier that aims to identify an optimal separating hyperplane between classes. Depending on the selected kernel, SVM can model either linear or nonlinear decision boundaries [19]. Together, these four classifiers enable the comparison of neural-network-based, ensemble-based, probabilistic linear, and margin-based learning approaches under the same validation conditions.

Furthermore, reflecting on the previously identified gaps in predicting cross-immunity and leveraging the available ML tools, we progressively expanded the physicochemical information content of the training set. It started with basic sequence data representation, extended it using amino acid similarity and conventional molecular descriptors, and ultimately incorporated the pharmacophore representation of epitopes. Here, a simple amino acid sequence contains only the order of amino acids, whereas similarity matrices, such as a block substitution matrix—position-weighted BLOSUM62 (“p.w.BLOSUM62”) [18], not only contains the sequence alignment of peptides but also the weight of importance of the position of the amino acids. Another similarity matrix that is applied is a point accepted mutation (PAM) matrix, such as “PAM250” [20], where the likelihood of the amino acid being replaced by another number based on observed substitutions in strictly homologous sequences within a given evolutionary period for proteins. Pharmacophore approaches are simplified 3D geometrical representation methods that identify the essential structural key points responsible for the biological activity of molecules (e.g. the position of hydrogen bond donors/acceptors, hydrophobic regions, and charged groups) [21]. The application of these key structural features enables the identification of novel peptides with similar activities even if their overall sequences differ significantly.

Finally, validation on an independent dataset indicated that incorporating amino acid similarity alone enhanced the predictive performance. In contrast, the limited contribution of structural features suggests that geometrical information alone may be insufficient to fully capture the complexity of TCR–epitope interactions. Thus, these results demonstrate that our strategy enables computational classification of structurally diverse epitopes with shared functional properties.

## Materials and methods

### Data collection and preprocessing

A comprehensive dataset was collected and processed from the literature, where the immune activity scores of 9-mer epitopes were presented for the HLA-A*0201 protein [12, 13]. Epitopes from the internal database were categorized as active or inactive using a threshold of moderate relative activation of 0.5 and applied in the validation. To account for pronounced differences in measurement conditions among the participating laboratories in the case of the first and the second external test sets (for a detailed description for the first and second external test sets see the Results and Discussion section), the relative activation threshold was increased to 0.7. In the second external test set, this threshold was close to the upper quartile of the response distribution (Q3 ≈ 0.693), which indicates careful selection of the thresholds. Thus, the 0.5 threshold preserved the original moderate activation definition in the training-related datasets, whereas the 0.7 threshold in external test sets defined a stricter, distribution-supported class of strongly activating peptides. All epitope sequences endowed with experimental immune activation values were used to generate peptide–MHC protein complexes using the AlphaFold3 server [22]. To generate the ligand–target complex, an experimental MHC-I crystal structure was applied using PDB code 3UTQ [23]. The resulting complexes were preprocessed using the Protein Preparation Wizard module of the Schrödinger Suite [24]. This included the determination of the protonation states at 7.4 pH and relaxation with 0.3 Å root mean square deviation (RMSD) criteria for heavy atoms.

### Molecular representations

**Figure 1:**
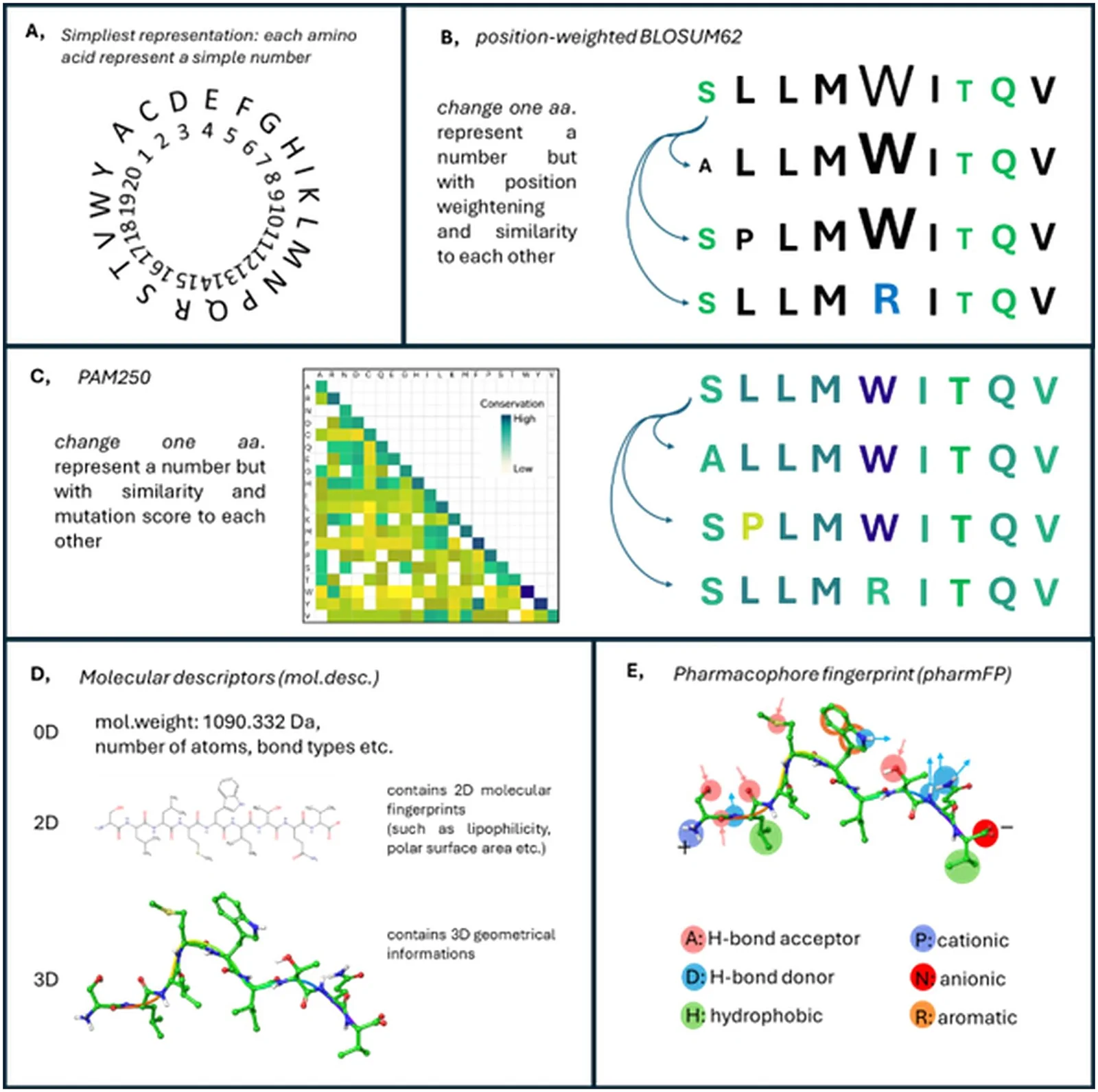
Numerical representation types of 9-mer epitopes; (a) simple amino acid sequence (aa.seq.); (b) similarity-matrix-based encoding using position-weighted BLOSUM62 (p.w.BLOSUM62); (c) mutation matrix-based encoding using PAM matrix (PAM250); (d) multiple molecular descriptors based on physicochemical properties such as chemical formula, molecular weight, bond angles, surface characteristics, etc. (mol.desc.); (e) 3D pharmacophore fingerprint based on annotated secondary interactions of the ligand (pharmFP); the epitope–protein complex 3D structures were generated by AlphaFold3 [22].

The numerical representation of the 9-mer epitopes with simple amino acid sequences is based on a one-to-one mapping, as shown in Fig. 1a, while the theoretical background of the other representations, such as similarity matrices and structural descriptors, is summarized in Fig. 1b–e. Thus, five different input datasets (representations) were generated for the concerned peptide sequences, namely “aa.seq.,” “p.w.BLOSUM62,” and “PAM250” [18, 20, 25], as well as traditional molecular descriptors (“mol.desc.”) [26] or Schrödinger’s pharmacophore features (“pharmFP”) [21, 27]. The applied similarity matrices are essential tools for quantifying sequences based on amino acid similarities. Molecular descriptors are direct or derived properties of a molecule that can be understood by assuming that molecules with similar descriptors have similar properties. The pharmacophore properties (H-bond donor/acceptor relation, charge, aromaticity, and hydrophobicity) include all the structural or chemical properties of a compound responsible for ligand binding according to the Canvas module in the Schrödinger suite [27]. For the molecular descriptors, we considered the complexes generated by the AlphaFold3 server [22] and used a set of 0D–3D descriptors of the epitopes produced by the Dragon software [24]. The generated molecular descriptors were unique to the peptides and were based on their 0D, 1D, 2D, and 3D features, resulting in ~3000 descriptors.

Pharmacophore fingerprints were generated using Canvas, a part of the Schrödinger Suite [25]. These pharmacophore fingerprints are also unique and are based on the three-point model used by Canvas on the Schrödinger platform, which yields ~10 000 data points.

### Model architectures

#### Feedforward neural network

**Figure 2:**
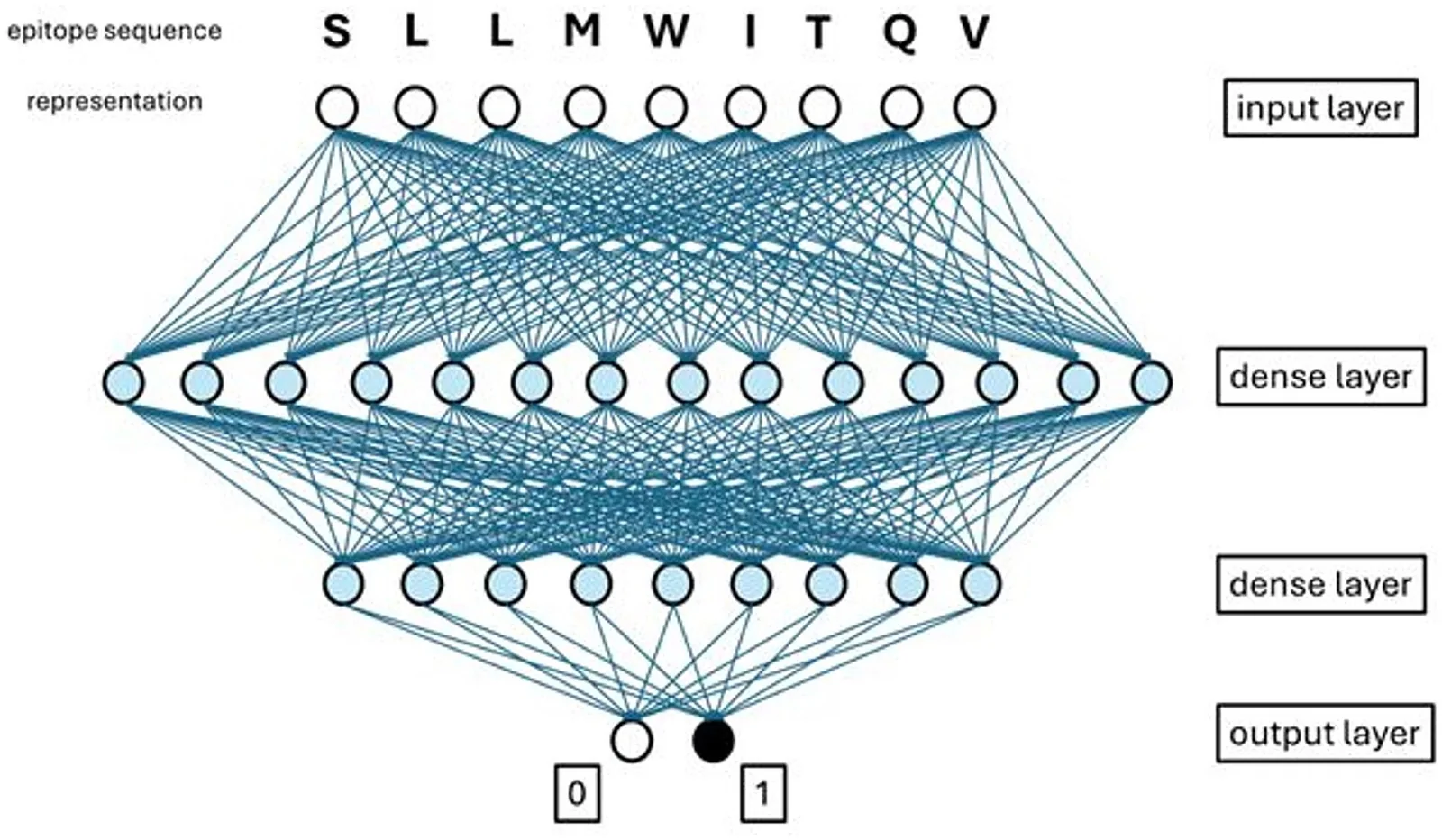
The collected 9-mer peptides are presented in selected representation in the input layer and subsequently classified binary as active or inactive epitopes (output layer) using a FFNN algorithm; according to the FFNN method, information flows unidirectionally without feedback loops.

In our study, we constructed a feed-forward artificial neural network in which information moves strictly in one direction (Fig. 2). Detailed information on the multilayer perceptron architectures, as well as the other models, can be found in Supplementary Table S1.

In our work, the 135-epitope dataset was divided into training and validation sets (internal test set) using 70%–30% splits to maintain a robust evaluation framework. The classification method was applied with five-fold randomized cross-validation during five loops of ML training [28]. A neural-network-based modeling workflow was created in the KNIME environment using the KERAS neural network package (see Supplementary Fig. S1) [29].

#### Random forest decision tree algorithm

RF is an ensemble learning method that is used for classification and regression models. This algorithm combines the outputs of multiple decision trees to obtain a single result. The basic idea of the RF is to construct multiple individual decision trees during the training phase and aggregate their predictions using a simple voting scheme. In this way, the final outcome is strengthened, and a single predicted value is obtained for each observation in the dataset [15].

#### Logistic regression algorithm

LR is a traditional supervised linear ML algorithm used primarily for classification tasks. It calculates a linear combination of input features and passes the raw output through a sigmoid (logistic) function. It is a commonly used baseline model because it is simple and can be trained quickly; however, it struggles to model complex, non-linear relationships without feature engineering, and it can only be used for classification tasks [16, 30].

#### Support vector machine

The SVM focuses on the data points that are difficult to classify (called support vectors) and draws a decision boundary that maximizes the gap between them. The major idea of SVM is that it defines a hyperplane in a higher-dimensional space to separate groups of samples. SVM models can be easily overfitted, because of several optimizable parameters and kernel functions; therefore proper validation is needed. On the other hand, SVM can handle complex, non-linear relationships, too. Unlike neural networks, which can be trapped in a local optimum, an SVM relies on convex optimization to find a globally optimal solution during training. Similar to NNs, SVMs can be slow and memory-extensive; however, they are generally not recommended for massive dataset analysis with hundreds of thousands of samples [17].

In the present study, the FFNN, RF, LR, and SVM methods were selected because of their broad applicability to classification tasks and their ability to handle different types of input features. These models are suitable and manageable for small datasets, owing to their simplicity. The results of the hyperparameter optimization for each artificial intelligent (AI) model are shown in Supplementary Table S1. To test the possibility of overfitting owing to the small number of training samples, we performed a permutation test with shuffling of the class labels (label shuffling), as detailed in Supplementary Table S2.

The developed ML models were implemented using KNIME software, as presented in Supplementary Fig. S1 [29].

### Consensus generation

The consensus function is defined as the average of the predicted class probabilities (from the output layer) across the selected models [31]. We applied the consensus method separately to combine the similarity matrix-based models (“p.w.BLOSUM62” with “PAM250”) and the structural descriptor-based versions (“mol.desc.” with “pharmFP”). Finally, we constructed a consensus function by equally weighting the results of the four representations.

### Evaluation metrics

To qualify the outcomes, binary classification with the most frequently applied evaluation metrics is presented. The simplest but most common method is *accuracy* which expresses the percentage of correctly classified instances among the members of the full set. The *sensitivity* value quantifies truly positive instances, whereas the *specificity* value expresses truly negative instances. In the literature, *sensitivity* is often referred to as *recall*. The formulas applied to these quantities are as follows [32]:


(1) \begin{equation*} Accuracy=\mathrm{ACC}.=\frac{TP+ TN}{TP+ TN+ FP+ FN}\kern1.75em \in \left[0,1\right]\kern0.75em \end{equation*}

(2) \begin{equation*} Sensitivity=\mathrm{Sen}.=\frac{TP}{TP+ FN}\kern1.75em \in \left[0,1\right] \end{equation*}

(3) \begin{equation*} Specificity=\mathrm{Spe}.=\frac{TN}{TN+ FP}\kern1.75em \in \left[0,1\right] \end{equation*}

True positive (TP), true negative (TN), false positive (FP), and false negative (FN) refer to the number of predictions using these designations. It should be noted that *sensitivity* and *specificity* reveal more about the model than *accuracy*, especially when the numbers of real positive and negative instances are unbalanced. Furthermore, we calculated additional evaluation metrics, such as *balanced accuracy* which depends on all the correctly classified values of the confusion matrix [32]. It assigns equal weight to the performance in all classes, provides a more reliable assessment of performance, and provides a balanced picture.


(4) \begin{equation*} Balanced\ accuracy=\mathrm{bACC}.=\frac{Sen.+ Spe.}{2}\kern1.75em \in \left[0,1\right] \end{equation*}

The F1-score is an important evaluation metric that measures the harmonic mean of *precision* and *sensitivity*, as defined by Van Rijsbergen [33].


(5) \begin{equation*} \mathrm{F}1-\ \mathrm{score}=\frac{2\ast Pre.\ast Sen.}{Pre.+ Sen.} \in \left[0,1\right] \text{ where } \kern0.75em \mathrm{Pre}.=\frac{TP}{TP+ FP} \in \left[0,1\right] \end{equation*}

while “Cohen’s kappa” (κ) is defined by McHugh [34] as follows:


(6) \begin{align*} &\mathrm{\kappa} =\frac{ACC.-{p}_e}{1.-{p}_e} \in \left[-\infty, 1\right] \text{ where }\notag\\ &{p}_e=\frac{\left( TP+ FN\right)\left( TP+ FP\right)+\left( TN+ FP\right)\left( TN+ FN\right)}{{\left( TP+ TN+ FP+ FN\right)}^2} \end{align*}

The latter compares the performance of the binary classifier with that of random accuracy (ACC) *p_e_*. Cohen’s κ is a quantity with a probability-based correction that helps filter biases that arise when a model seems to perform well owing to an unbalanced distribution or according to ACC, but it is no better than a random guess. It can be applied to measure the agreement between predicted and real classes.

Another typical performance metric is the receiver operating characteristic (ROC) curve, which is a visual representation of performance [35]. This demonstrates the model’s ability to distinguish between positive and negative classes across all possible classification thresholds. The perfect model shows a square with unit sides and an area under the curve (AUC) of 1.0. This implies that it has 100% probability of a randomly selected positive case that will be correctly categorized by the model at a continuously varying threshold value.

## Results and discussion

Variations in T-cell activation may arise from structural differences among interacting peptides, which in turn modulate the molecular surface of epitope–MHC-I complexes. Since the sequence and primary geometry of the MHC-I protein remain constant, it is a trivial assumption that the chemical properties of the epitope are the key points that predominantly determine the formation of the complex and subsequent T-cell recognition. Based on the response of T-cells activated by the pMHC complexes formed, we classified the peptides as active or inactive (see Methods). A peptide was considered active if it was capable of retaining its specific biological functions, such as TCR binding or T-cell activation. Although these two effects are not independent of each other, the applied experimental biological data typically address only one of them at a time. Furthermore, to provide a cost-effective AI-driven workflow, we incorporated information from computationally generated 3D structures of epitope–MHC complexes, as detailed in the Methods section. Thus, diverse representations of epitopes could be explored, ranging from simple amino acid sequences to similarity matrix-derived scores or 3D structural characteristics. By integrating these distinct layers of molecular information, including peptide sequence-based information (“aa.seq.,” “p.w.BLOSUM62,” “PAM250”) or molecular descriptors (“mol.desc.”) and pharmacophore features (“pharmFP”), a comprehensive predictive framework was constructed.

In principle, neural networks can perform robustly on relatively small datasets, as their architecture helps limit overfitting, but their large number of parameters allows them to memorize the noise [36]; however, decision trees are generally robust to overfitting, whereas in small-sample regimes, the bootstrap samples used to train individual trees can exhibit substantial overlap. Probabilistic methods can capture the noise in the training data rather than underlying patterns which leads to poor generalization, whereas margin-based methods can push the margin to favor the majority class while failing to correctly classify the minority, as in an imbalanced dataset.

**Figure 3:**
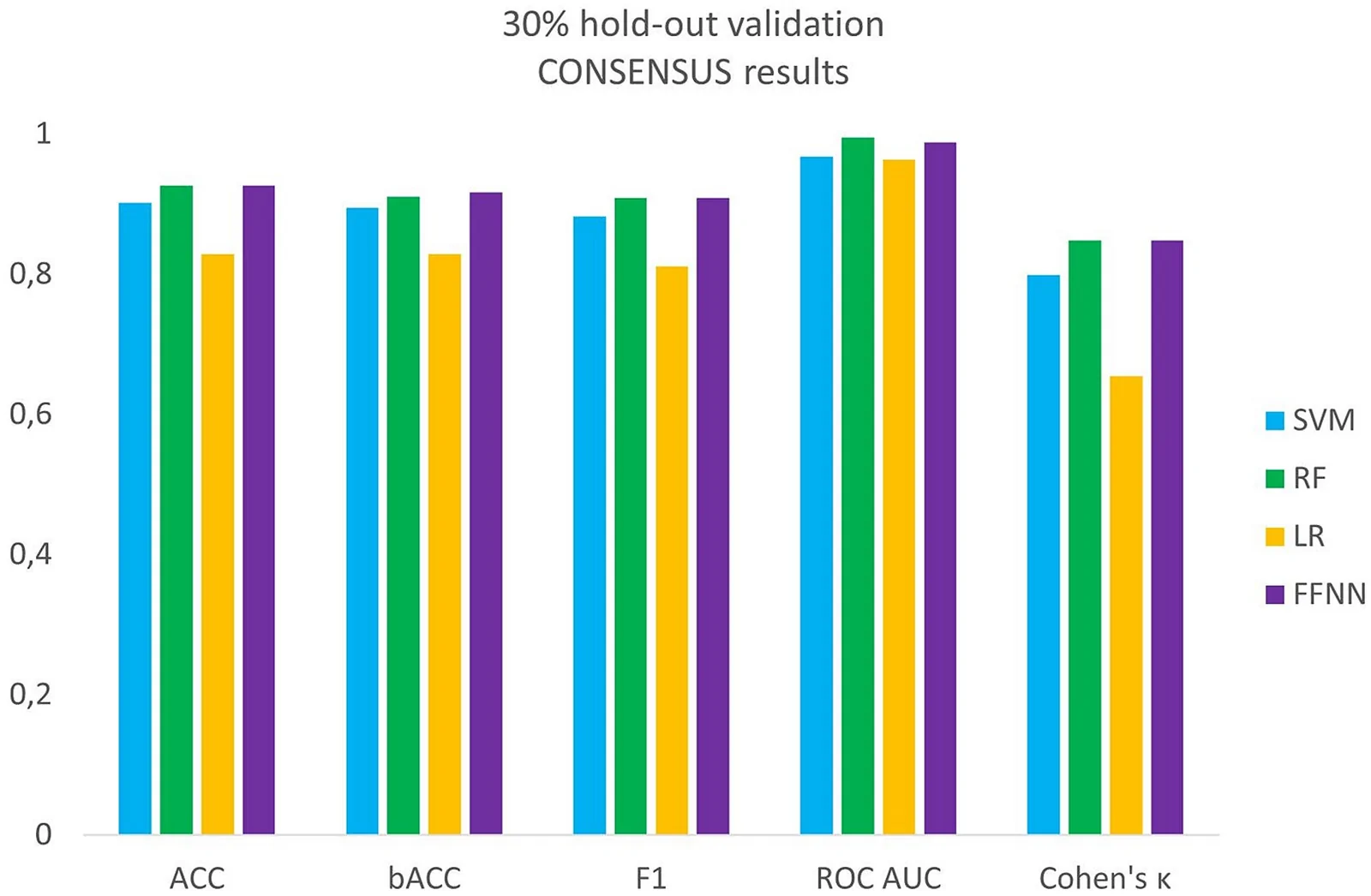
Qualitative attributes of 30% hold-out (internal) validation test, used different ML models (SVM, LR, RF, and the FFNN) across consensus function of different epitope representations; the qualitative descriptors are: ACC = accuracy, bACC = balanced accuracy, ROC AUC = receiver operating characteristic area under the curve; exact numerical values can be found in Supplementary Table S3.

In our model training, the 135-epitope dataset was randomly divided into a 70% training subset and 30% hold-out (internal) validation subset. Model optimization was performed on the training subset using five-fold cross-validation, and the final performance was evaluated on the independent 30% hold-out set (Fig. 3). Cross-validation checks were carried out (see Supplementary Fig. S2), and the partial results for the individual representations are shown in Supplementary Fig. S3. In the main text, we present advanced consensus function results based on similarity matrices (“p.w.BLOSUM62” + “PAM250”) and structural descriptors (“mol.desc.” + “pharmFP”) (see details in the Methods section). The “aa.seq.” representation type was not included in the consensus function because of its poor performance (see Supplementary Fig. S3).

The quality of the methods was represented by five selected performance parameters: ACC, balanced accuracy (bACC), Cohen’s κ, ROC AUC, and F1-score, whose descriptions are described in the “Methods” section.

The consensus-based hold-out (internal) validation results (Fig. 3) showed strong overall performance for all four classifiers across the 30% validation set. RF and FFNN achieved the most consistent performance, with ACC, bACC, and F1-score values of ~0.90 or higher, indicating a robust and well-balanced classification of both active and inactive epitopes. SVM also performed well, particularly in terms of the ROC AUC, although its Cohen’s κ was slightly lower than that of RF and FFNN. LR showed the weakest overall performance among the four models, especially for the F1-score and Cohen’s κ, suggesting a reduced ability to capture the more complex relationships present in the combined peptide representations. Overall, the consensus approach produced high ROC AUC values for all classifiers, indicating that the combined input representations retained highly discriminative information. These results underline the preferred application of multiple complementary classifiers, rather than relying exclusively on a single modeling strategy. We would like to note that for completeness the results of the corresponding single models can be found in the Supplementary Fig. S3.

**Figure 4:**
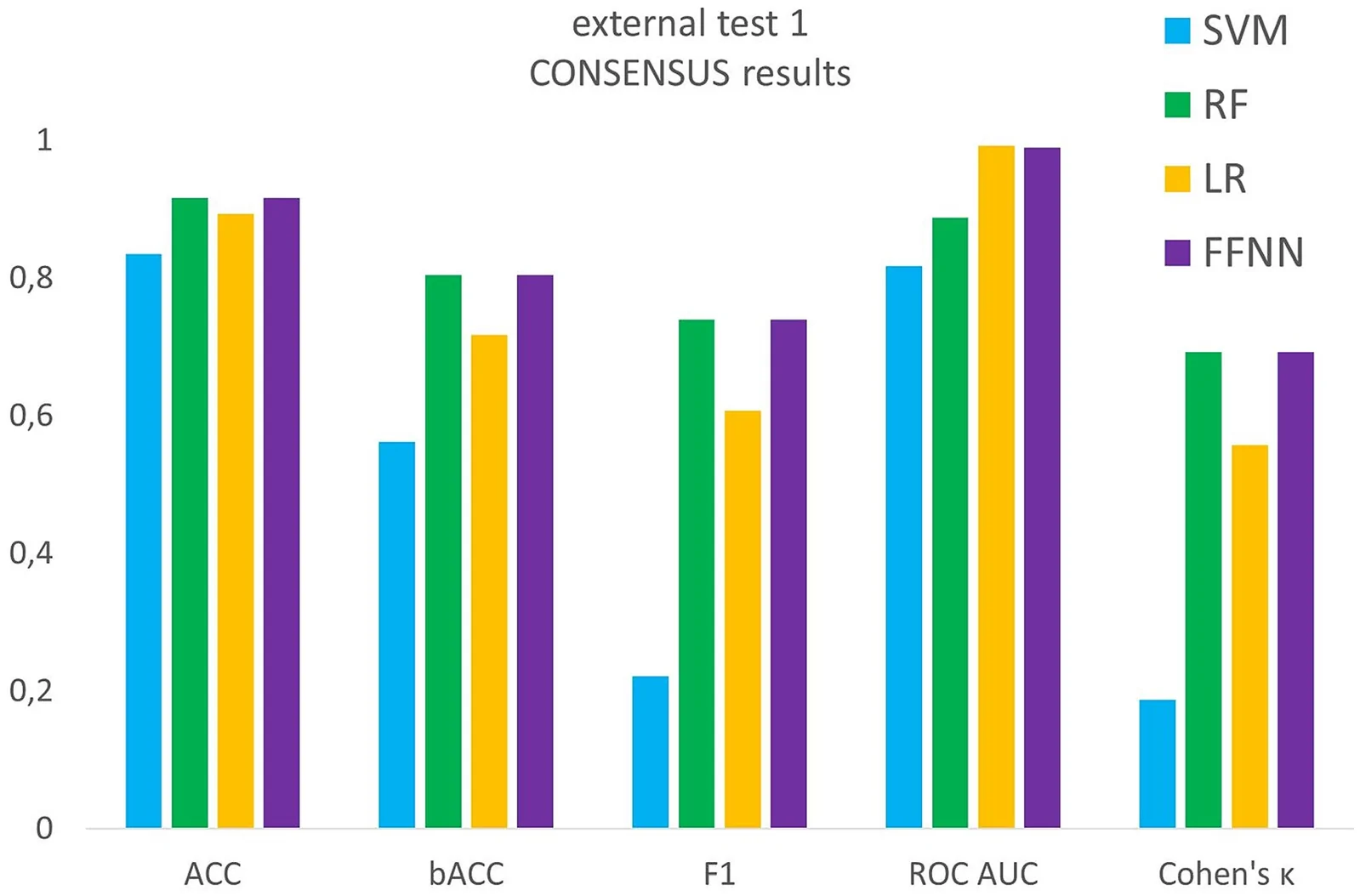
Performance metrics for the first external test set evaluated using different AI models (SVM, LR, RF, and the FFNN) across consensus function; the results are based on the classification of a dataset comprising 85 additional, previously unseen epitopes originating from the same research group that generated the initial 135-epitope dataset; the qualitative descriptors are: ACC = accuracy, bACC = balanced accuracy, ROC AUC = receiver operating characteristic area under the curve; exact numerical values can be found in Supplementary Table S4.

An important aspect of the first results is that, although the FFNN and RF methods achieved good performance during internal validation, the main challenge of AI methods remains their applicability to external datasets. Therefore, external validation and comparative analysis were performed using the full set of previous samples (135 epitopes) as the training data. During this phase, the same AI methodologies as those in the initial training and validation stages were applied, and classification was conducted on an independent external dataset comprising 85 previously unseen epitopes. Notably, the measurement data for the new set of epitopes were provided by the same research group using the same experimental techniques. The resulting consensus descriptor values are shown in Fig. 4.

The first external test set supported the transferability of the consensus-based approach although clear classifier-dependent differences were observed. RF and FFNN showed the most consistent performance across threshold-dependent metrics, including bACC, F1-score, and Cohen’s κ, suggesting a more balanced classification of activating and non-activating epitopes. LR achieved the highest ROC AUC, indicating a strong discriminative ranking of peptide activity probabilities; however, its lower F1-score and Cohen’s κ suggest that this ranking performance did not fully translate into an optimal binary classification. The SVM also showed good overall ACC, but its weaker F1-score and Cohen’s κ indicated reduced performance in the identification of the active class. Together, these results suggest that the consensus input strategy remains informative in an independent external validation setting, with RF and FFNN providing the most balanced classification profiles.

**Figure 5:**
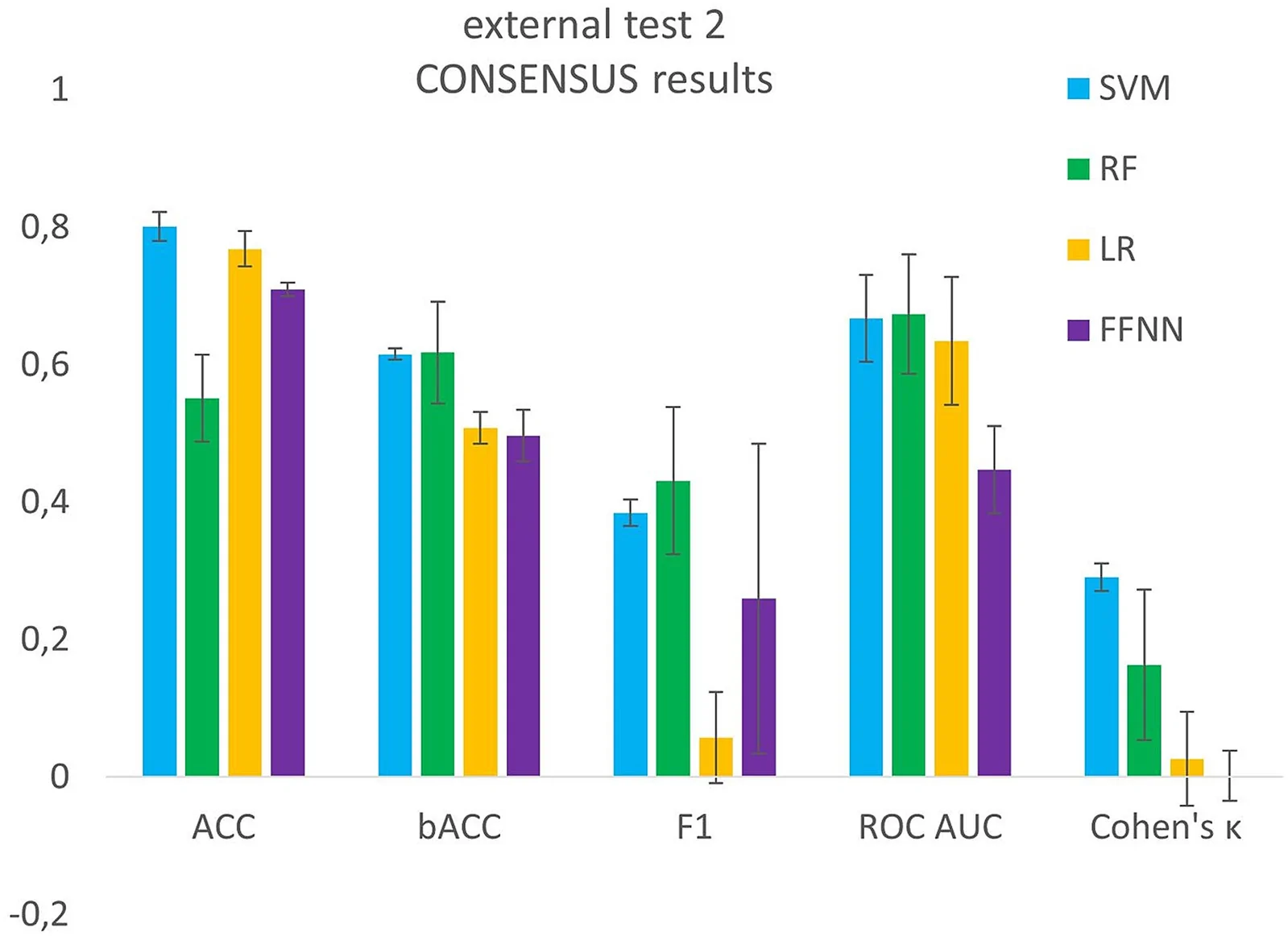
Performance metrics for the second external test set evaluated using different AI models: SVM, LR, RF, and the FFNN across consensus function; the second external test set comprises the TCR1, TCR2, and TCR3 cell lines, and the figure presents mean values across these datasets, with SDs shown above the columns; the abbreviation for descriptors means: ACC = accuracy, bACC = balanced accuracy, ROC AUC = receiver operating characteristic area under the curve; exact values can be found in Supplementary Table S5.

To further assess the robustness and cross-dataset generalizability of the models, we evaluated their performance on a second independent external dataset (second external test set), and the results are presented in Fig. 5. The second external test set contains 172 additional epitopes reported by different research groups [13] and measured in three altered TCR-defined T-cell populations, referred to as TCR1, TCR2, and TCR3. These populations represent distinct T-cell lineages, with TCR1 corresponding to the avian equivalent of mammalian γδ T-cells, whereas TCR2 and TCR3 represent two αβ T-cell lineages. Therefore, this dataset provided a more stringent validation scenario, allowing us to test whether predictive performance (observed for the first external test set) could be maintained across biologically distinct TCR response contexts.

Therefore, the second external test set represents a more challenging validation scenario, as reflected by the lower and more variable performance across the classifiers. The SVM showed the most favorable overall profile, achieving the highest ACC and Cohen’s κ, together with moderate bACC and ROC AUC. RF provided comparable bACC and the highest F1-score, suggesting a relatively better ability to identify activating epitopes, although its overall ACC and Cohen’s κ were lower than those of the SVM. LR achieved a high ACC but showed a markedly reduced F1-score and Cohen’s κ, indicating that its performance was strongly influenced by the dominant class and that it was less effective in detecting the active class. The FFNN showed the weakest overall performance on this external dataset, with lower ACC and ROC AUC values, suggesting limited generalization under this more heterogeneous external validation setting. Overall, these results indicate that the second external test set was substantially more difficult to classify than the previous ones (internal hold-out and first external test set), highlighting the impact of diverse laboratory conditions, activity definitions, and experimental variability on cross-dataset model transferability. The discrepancy between the ACC and F1-score, particularly for LR, indicates that ACC alone is insufficient for evaluating performance on this second external dataset, and that balanced metrics are required to assess active-class recognition.

Considering all the results obtained, we found that for the internal validation case, the FFNN and RF methods provided the most consistent performance across all metrics. Challenging the model using external datasets, the results of external tests did not support the universal superiority of a single classifier across all datasets. Instead, they indicate that the model performance was dependent on the external dataset and evaluation metrics. In the case of the first external test set, RF and FFNN showed the most balanced classification profiles, with strong ACC, bACC, F1-score, ROC AUC, and Cohen’s κ values. This suggests that both nonlinear approaches can generalize well to this independent validation set. However, the second external test represents a more challenging validation scenario. In this latter situation, SVM and LR achieved higher overall ACC, but RF retained competitive bACC, F1-score, and ROC AUC, indicating a better ability to identify the active class than ACC alone. In contrast, the FFNN showed weaker model transferability on this dataset, whereas RF appeared to provide consistent nonlinear performance across both external datasets. The consensus strategy was intentionally included to evaluate whether complementary information from different peptide representations could improve robustness compared with relying on a single representation. Each representation captures a different aspect of the peptide sequence or structure: substitution matrix similarity, physicochemical/structural descriptors, and pharmacophore-related information. As these representations may produce different errors, averaging their predicted probabilities can reduce representation-specific noise and partially compensate for individual model weaknesses.

## Conclusion

In conclusion, our findings do not substantiate the universal superiority of any single classifier across all validation settings. Instead, they indicate that the FFNN, RF, LR, and SVM provide complementary modeling perspectives in different external test scenarios. RF offered stable nonlinear performance across the two external datasets, whereas the FFNN performed well on the hold-out (internal test set) and on a similar external laboratory test case (first external test set), but was less robust on the more distinct external test (second external test set). These results support the application of consensus-based representations in combination with multiple complementary classifiers, and underscore the importance of independent external validation and balanced performance metrics when evaluating TCR-related epitope activation models. Therefore augmenting the current approach with experimental measurements will open new directions for the investigation of cross-immunity.

**Key Points**

- Different sets of 9-mer epitopes endowed with literature-based experimental Human Leukocyte Antigen (HLA) activation data were represented in five different ways.
- Feedforward neural networks, random forest, logistic regression, and support vector machine algorithms were applied to develop binary classification models to predict HLA activation ability for each representation type.
- Using five different qualitative descriptors, we evaluated the performance of artificial intelligent models for each representation, including the application of different consensus models.

## Supplementary Material

AcceptedManuscript-BIB-26-0805-SuppInf_bbag484

## Data Availability

All relevant data can be found in the paper and its Supporting Information and in the Github repository located at https://github.com/reschphd-code/ML-crossimmu.git.
