# Supplementary material for: Machine learning-based prediction of cross-immunity
Source: Brief Bioinform. 2026 Sep 10;27(5):bbag484. doi: 10.1093/bib/bbag484 (PMC13561412; doi:10.1093/bib/bbag484)
Supplement: Supplementary file 1 [file acceptedmanuscript-bib-26-0805-suppinf_bbag484.docx]

**Supporting Information**

**Machine Learning-based prediction of Cross Immunity**

Vivien Erzsébet Resch^1,2^, László Tóth ^3^, Anita Rácz ^4,^*, Dezső Virok^5,^*, Gábor Paragi^1,2,6^ *

^1^Department of Medicinal Chemistry, University of Szeged, Dóm tér 8, H-6720 Szeged, Hungary

^2^Institute of Physics, University of Pécs, Ifjúság útja 6, H-7625 Pécs, Hungary

^3^ Institute of Informatics, University of Szeged, Árpád tér 2, 6720 Szeged, Hungary

^4^Plasma Chemistry Research Group, HUN-REN Research Centre for Natural Sciences, Magyar Tudósok Körútja 2, Budapest, H-1117, Hungary

^5^Department of Medical Microbiology, Albert Szent-Györgyi Health Center and Albert Szent-Györgyi Medical School, University of Szeged, Semmelweis Str. 6, H-6725 Szeged, Hungary

^6^ Department of Theoretical Physics, University of Szeged, Tisza L. krt. 84-86, H-7625 Pécs, Hungary

* Corresponding authors.

Gábor Paragi, (ORCID: [0000-0001-5408-1748](https://orcid.org/0000-0001-5408-1748))
Tel.: +36-72-503-600/24399, Fax: +36-72-501-571, E-mail: [paragi@gamma.ttk.pte.hu](mailto:paragi@gamma.ttk.pte.hu)

Dezső Virok, (ORCID: [0000-0002-9456-7993](https://orcid.org/0000-0002-9456-7993))
Tel: +36-62-545-541, Fax: +36-62-546-371, E-mail: [virok.dezso.peter@med.u-szeged.hu](mailto:virok.dezso.peter@med.u-szeged.hu)

Anita Rácz, (ORCID: 0000-0001-8271-9841)
Tel: +36-1-3826-509, Fax: +36-1-382-6500, E-mail: [racz.anita@ttk.hu](mailto:racz.anita@ttk.hu)

**Results of the algorithms**

**SI Table 1. Hyperparameter optimized ML models (using grid-search).**

| SVM | **aa.seq.** | **p.w.BLOSUM62** | **PAM250** | **mol.desc.** | **pharmFP** |
| --- | --- | --- | --- | --- | --- |
| class weight | balanced | none | none | none | balanced |
| C | 10 | 10 | 1 | 100 | 10 |
| gamma | auto | scale | scale | scale | auto |
| kernel | rbf | rbf | linear | linear | rbf |

| RF | **aa.seq.** | **p.w.BLOSUM62** | **PAM250** | **mol.desc.** | **pharmFP** |
| --- | --- | --- | --- | --- | --- |
| class weight | balanced | none | none | balanced | balanced |
| max. depth | 10 | 10 | none | 5 | 5 |
| max. features | none | none | none | log2 | none |
| estimators | 100 | 100 | 100 | 200 | 500 |

| LR | **aa.seq.** | **p.w.BLOSUM62** | **PAM250** | **mol.desc.** | **pharmFP** |
| --- | --- | --- | --- | --- | --- |
| class weight | balanced | balanced | none | balanced | balanced |
| C | 1 | 1 | 1 | 1 | 10 |
| penalty | l1 | l2 | l2 | l2 | l2 |

| FFNN | **aa.seq.** | **p.w.BLOSUM62** | **PAM250** | **mol.desc.** | **pharmFP** |
| --- | --- | --- | --- | --- | --- |
| hidden layers | 100,50 | 100,50 | 16,8 | 100,50 | 100,50 |
| activation layer | relu | relu | relu | relu | relu |
| learning rate | 0.003 | 0.003 | 0.003 | 0.0005 | 0.001 |
| l2 | 0.0 | 0.0 | 0.001 | 0.001 | 0.01 |
| drop out | 0.0 | 0.2 | 0.0 | 0.2 | 0.2 |
| batch | 15 | 5 | 15 | 10 | 8 |
| epoch | 10 | 5 | 5 | 10 | 15 |
| optimizer | adam | adam | adam | adam | adam |
| loss function | binary cross entropy | binary cross entropy | binary cross entropy | binary cross entropy | binary cross entropy |
| random seed | 42 | 42 | 42 | 42 | 42 |

**SI Figure 1.** Map of the applied Machine Learning workflow. Started with data processing of each input type. Then the training and validation is made to choose the best tools. Whole dataset is used for the training to challenge our models with an external test, using KNIME software [1].

**SI Figure 2.** Mean of the qualitative descriptors of the 5-fold cross-validation for different epitope representations on the applied ML models (Support Vector Machine (SVM), Logistic Regression (LR), Random Forest (RF) and the Feed Forward Neural Network (FFNN)). Training dataset consists randomly selected 70% of 135 epitopes and tested in each CV iteration. The applied representations of the epitopes: **aa.seq** = amino acid sequence, **p.w.BLOSUM62** = position weighted BLOSUM62 matrix, **PAM250** = PAM250 percent accepted mutation matrix, **mol.desc**.= molecular descriptors, **pharmFP** = pharmacophore fingerprints used as numerical representations of epitopes). ACC = accuracy, bACC = balanced accuracy, ROC AUC = receiver operating characteristic area under the curve.

**SI Table 2.** Results of the permutation tests across **bACC** (balanced accuracy) with the applied ML models (Support Vector Machine (SVM), Logistic Regression (LR), Random Forest (RF) and the Feed Forward Neural Network (FFNN) on the **30% hold-out (internal) validation** set. Observed value = internal validation accuracy, shuffle mean = the prediction results after randomly 20 times shuffled label and shuffle std = standard deviation.

|  | **RF** | | | **LR** | | | **SVM** | | | **FFNN** | | |
| --- | --- | --- | --- | --- | --- | --- | --- | --- | --- | --- | --- | --- |
|  | observed value | shuffle mean | shuffle SD | observed value | shuffle mean | shuffle SD | observed value | shuffle mean | shuffle SD | observed value | shuffle mean | shuffle SD |
| **aa.seq.** | 0.913 | 0.513 | 0.139 | 0.691 | 0.498 | 0.103 | 0.808 | 0.505 | 0.121 | 0.721 | 0.514 | 0.107 |
| **p.w.BLOSUM62** | 0.917 | 0.494 | 0.110 | 0.879 | 0.484 | 0.150 | 0.879 | 0.486 | 0.162 | 0.917 | 0.517 | 0.123 |
| **PAM250** | 0.944 | 0.502 | 0.110 | 0.901 | 0.501 | 0.143 | 0.873 | 0.485 | 0.141 | 0.836 | 0.518 | 0.132 |
| **mol.desc.** | 0.802 | 0.504 | 0.089 | 0.879 | 0.508 | 0.087 | 0.774 | 0.508 | 0.097 | 0.839 | 0.500 | 0.120 |
| **pharmFP** | 0.818 | 0.490 | 0.083 | 0.824 | 0.524 | 0.079 | 0.845 | 0.491 | 0.056 | 0.901 | 0.491 | 0.078 |

**SI Figure 3.** **30% hold-out (internal) validation test** results in the applied models (Support Vector Machine (SVM), Logistic Regression (LR), Random Forest (RF) and the Feed Forward Neural Network (FFNN)). The applied representations of the epitopes: **aa.seq** = amino acid sequence**, p.w.BLOSUM62** = position weighted BLOSUM62 matrix, **PAM250** = PAM250 percent accepted mutation matrix, **mol.desc.**= molecular descriptors, **pharmFP** = pharmacophore fingerprints used as numerical representations of epitopes). ACC = accuracy, bACC = balanced accuracy, ROC AUC = receiver operating characteristic area under the curve.

**SI Table 3.** Qualitative attributes of **30% hold-out (internal) validation test**, used different ML models (Support Vector Machine (SVM), Logistic Regression (LR), Random Forest (RF) and the Feed Forward Neural Network (FFNN)) across consensus function of different epitope representations. Dataset is represented as: similarity matrices (**p.w.BLOSUM62** = position-weighted BLOSUM62 matrix + **PAM250** = point accepted mutation matrix) and as structural descriptors (**mol.desc**.= molecular descriptors + **pharmFP** = pharmacophore fingerprints)), while qualitative descriptors are: ACC = accuracy, bACC = balanced accuracy, ROC AUC = receiver operating characteristic area under the curve

| **SVM** | **aa.seq.** | **p.w.BLOSUM62** | **PAM250** | **mol.desc.** | **pharmFP** |
| --- | --- | --- | --- | --- | --- |
| **ACC** | **0.781** | **0.902** | **0.878** | **0.829** | **0.878** |
| **bACC** | **0.756** | **0.895** | **0.861** | **0.823** | **0.879** |
| **F1** | **0.689** | **0.882** | **0.838** | **0.800** | **0.864** |
| **ROC AUC** | **0.804** | **0.990** | **0.899** | **0.898** | **0.905** |
| **Cohen’s κ** | **0.530** | **0.779** | **0.744** | **0.651** | **0.754** |

| **RF** | **aa.seq.** | **p.w.BLOSUM62** | **PAM250** | **mol.desc.** | **pharmFP** |
| --- | --- | --- | --- | --- | --- |
| **ACC** | **0.829** | **0.829** | **0.926** | **0.829** | **0.853** |
| **bACC** | **0.841** | **0.805** | **0.922** | **0.823** | **0.851** |
| **F1** | **0.829** | **0.758** | **0.914** | **0.651** | **0.833** |
| **ROC AUC** | **0.915** | **0.987** | **0.993** | **0.898** | **0.903** |
| **Cohen’s κ** | **0.663** | **0.638** | **0.850** | **0.800** | **0.702** |

| **LR** | **aa.seq.** | **p.w.BLOSUM62** | **PAM250** | **mol.desc.** | **pharmFP** |
| --- | --- | --- | --- | --- | --- |
| **ACC** | **0.658** | **0.902** | **0.901** | **0.819** | **0.853** |
| **bACC** | **0.677** | **0.888** | **0.888** | **0.823** | **0.851** |
| **F1** | **0.681** | **0.875** | **0.875** | **0.800** | **0.833** |
| **ROC AUC** | **0.722** | **0.990** | **0.996** | **0.900** | **0.891** |
| **Cohen’s κ** | **0.339** | **0.797** | **0.708** | **0.651** | **0.702** |

| **FFNN** | **aa.seq.** | **p.w.BLOSUM62** | **PAM250** | **mol.desc.** | **pharmFP** |
| --- | --- | --- | --- | --- | --- |
| **ACC** | **0.835** | **0.951** | **0.902** | **0.829** | **0.829** |
| **bACC** | **0.851** | **0.944** | **0.900** | **0.817** | **0.817** |
| **F1** | **0.833** | **0.941** | **0.888** | **0.787** | **0.787** |
| **ROC AUC** | **0.910** | **0.955** | **0.964** | **0.821** | **0.872** |
| **Cohen’s κ** | **0.702** | **0.961** | **0.801** | **0.646** | **0.646** |

**SI Table 4.** Qualitative attributes of **1^st^ external test**, used different ML models (Support Vector Machine (SVM), Logistic Regression (LR), Random Forest (RF) and the Feed Forward Neural Network (FFNN)) across consensus function of different epitope representations. The **external test 1** contains an additional 85 epitopes by the same research group. Dataset is represented as: similarity matrices (**p.w.BLOSUM62** = position-weighted BLOSUM62 matrix + **PAM250** = point accepted mutation matrix) and as structural descriptors (**mol.desc**.= molecular descriptors + **pharmFP** = pharmacophore fingerprints)) and combination of them: similarity matrices + structural descriptors, while qualitative descriptors are: ACC = accuracy, bACC = balanced accuracy, ROC AUC = receiver operating characteristic area under the curve.

| **SVM** | **similarity matrices** | **structural descriptors** | **combination of four components** |
| --- | --- | --- | --- |
| **ACC** | **0.835** | **0.850** | **0.835** |
| **bACC** | **0.562** | **0.625** | **0.562** |
| **F1** | **0.222** | **0.400** | **0.222** |
| **ROC AUC** | **0.994** | **0.761** | **0.818** |
| **Cohen’s κ** | **0.188** | **0.351** | **0.188** |

| **RF** | **similarity matrices** | **structural descriptors** | **combination of four components** |
| --- | --- | --- | --- |
| **ACC** | **0.505** | **0.847** | **0.917** |
| **bACC** | **0.623** | **0.593** | **0.805** |
| **F1** | **0.382** | **0.315** | **0.740** |
| **ROC AUC** | **0.877** | **0.809** | **0.888** |
| **Cohen’s κ** | **0.132** | **0.272** | **0.693** |

| **LR** | **similarity matrices** | **structural descriptors** | **combination of four components** |
| --- | --- | --- | --- |
| **ACC** | **0.929** | **0.870** | **0.894** |
| **bACC** | **0.812** | **0.656** | **0.718** |
| **F1** | **0.769** | **0.471** | **0.608** |
| **ROC AUC** | **0.994** | **0.906** | **0.993** |
| **Cohen’s κ** | **0.730** | **0.420** | **0.558** |

| **FFNN** | **similarity matrices** | **structural descriptors** | **combination of four components** |
| --- | --- | --- | --- |
| **ACC** | **0.811** | **0.905** | **0.917** |
| **bACC** | **0.836** | **0.822** | **0.805** |
| **F1** | **0.636** | **0.733** | **0.740** |
| **ROC AUC** | **0.973** | **0.961** | **0.990** |
| **Cohen’s κ** | **0.528** | **0.676** | **0.693** |

**SI Table 5.** Qualitative attributes ± SD of **2^nd^ external test** [2], used different ML models (Support Vector Machine (SVM), Logistic Regression (LR), Random Forest (RF) and the Feed Forward Neural Network (FFNN)) across consensus function of different epitope representations. The **external test 2** contains an additional 172 epitopes by different research group and tested on TCR1, TCR2 and TCR3 cell lines. The results are the mean of prediction results of that three TCR cell lines and their SD. Dataset is represented as: combination of similarity matrices matrices (**p.w.BLOSUM62** = position-weighted BLOSUM62 matrix + **PAM250** = point accepted mutation matrix) and structural descriptors (**mol.desc**.= molecular descriptors + **pharmFP** = pharmacophore fingerprints), while qualitative descriptors are: ACC = accuracy, bACC = balanced accuracy, ROC AUC = receiver operating characteristic area under the curve.

| **SVM** | **combination of four components** | **± SD** |
| --- | --- | --- |
| **ACC** | **0.801** | **0.021** |
| **bACC** | **0.615** | **0.008** |
| **F1** | **0.384** | **0.019** |
| **ROC AUC** | **0.668** | **0.063** |
| **Cohen’s κ** | **0.291** | **0.019** |

| **RF** | **combination of four components** | **± SD** |
| --- | --- | --- |
| **ACC** | **0.551** | **0.063** |
| **bACC** | **0.618** | **0.074** |
| **F1** | **0.431** | **0.104** |
| **ROC AUC** | **0.674** | **0.087** |
| **Cohen’s κ** | **0.163** | **0.109** |

| **LR** | **combination of four components** | **± SD** |
| --- | --- | --- |
| **ACC** | **0.769** | **0.026** |
| **bACC** | **0.508** | **0.023** |
| **F1** | **0.057** | **0.066** |
| **ROC AUC** | **0.535** | **0.093** |
| **Cohen’s κ** | **0.026** | **0.067** |

| **FFNN** | **combination of four components** | **± SD** |
| --- | --- | --- |
| **ACC** | **0.709** | **0.009** |
| **bACC** | **0.497** | **0.037** |
| **F1** | **0.260** | **0.225** |
| **ROC AUC** | **0.447** | **0.064** |
| **Cohen’s κ** | **0.002** | **0.036** |

**Supplementary References**

[1]	Berthold MR, Cebron N, Dill F, Gabriel TR, Kötter T, Meinl T, Ohl P, Sieb C, Thiel K, Wiswedel B. KNIME: The Konstanz Information Miner. In: Preisach C, Burkhardt H, Schmidt-Thieme L, Decker R, editors. Data Analysis, Machine Learning and Applications. Berlin, Heidelberg: Springer Berlin Heidelberg; 2008. p. 319–326.

[2]	Cai L, Caraballo Galva LD, Peng Y, Luo X, Zhu W, Yao Y, Ji Y, He Y. Preclinical Studies of the Off-Target Reactivity of AFP158-Specific TCR Engineered T Cells. Front Immunol. 2020;11. https://doi.org/10.3389/fimmu.2020.00607
